# Chrysophanol inhibits the osteoglycin/mTOR and activats NF2 signaling pathways to reduce viability and proliferation of malignant meningioma cells

**DOI:** 10.1080/21655979.2021.1885864

**Published:** 2021-02-23

**Authors:** Jiapeng Wang, Peng Lv

**Affiliations:** a Department of Intensive Medicine, Suizhou Hospital, Hubei University of Medicine, Suizhou, Hubei, China; b Department of Neurosurgery, Union Hospital, Tongji Medical College, Huazhong University of Science and Technology, Wuhan, Hubei, China;; c Department of Neurosurgery, Suizhou Hospital, Hubei University of Medicine, Suizhou, Hubei, China

**Keywords:** Chrysophanol, malignant meningioma, apoptosis, osteoglycin, mTOR, nf2

## Abstract

Chrysophanol shows promising antitumor activity, but how it may work against malignant meningioma is poorly understood. In addition, osteoglycin (OGN) may help mediate the antitumor effects of chrysophanol; thus, this study investigated the potential antitumor mechanism of chrysophanol in malignant meningioma cultures. Meningioma cell line HBL-52 were incubated with varying doses of chrysophanol (0–90 μM) for different time points, and osteoglycin (OGN) was overexpressed or inhibited in some cell cultures to assess its roles. Cell viability was quantified using the CCK8 assay and colony formation assays, while effects on cell cycle distribution and apoptotic rates were examined by flow cytometry and enzyme-linked immunosorbent assays (ELISA) to detect histone DNA levels. Caspase-3 and −9 activities were detected by related commercial kits. Protein expression was assessed using Western blotting. Chrysophanol significantly reduced HBL-52 cell viability, based on reduced colony formation, and proliferation, based on low levels of bromodeoxyuridine incorporation. Annexin V/propidium iodide staining revealed a 30% increase in apoptotic cells at 90 μM chrysophanol (33.7% vs 3.3% in control cultures). Chrysophanol treatment greatly decreased the Bcl-2/Bax expression ratio and increased the expressions of cleaved caspase-3 and −9, and the activities of caspase-3 and −9. Chrysophanol blocked cells in G1 phase and inhibited the OGN/mTOR signaling cascade, but activated neurofibromatosis 2 (NF2) cascade. OGN overexpression activated mTOR, down-regulated NF2, and partially reversed growth inhibition by chrysophanol. Chrysophanol may be useful as a treatment against malignant meningioma by inhibiting OGN/mTOR signaling and activating NF2 signaling.

## Graphical Abstract

**Figure:**
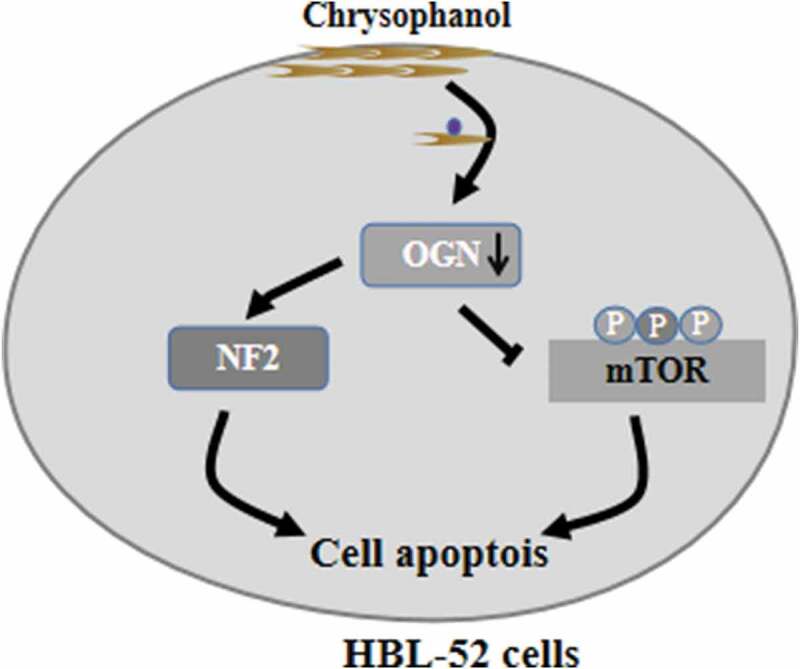

## Introduction

Meningiomas are the most common primary brain cancers in over the world [1]. Disease severity is classified into benign, atypical, and anaplastic/malignant, and 15 histologic subtypes have been identified [2]. Although most meningiomas are benign and grow slowly, malignant tumor frequently recurs and leads to the poor prognosis [3,4]. Therefore, more effective pharmacotherapeutic strategies are needed against meningiomas.

Osteoglycin (OGN) has important physiological roles in maintaining bone formation and normal vasculature [5–7], while its expression is associated with several diseases [7]. For example, OGN promotes meningioma cell proliferation by interacting with other drivers of tumor development, including neurofibromatosis 2 (NF2) and mammalian target of rapamycin (mTOR) [8]. Indeed, NF2 overexpression is associated with neurofibromatosis type 2, an inherited genetic disease involving meningioma [9]. High expression of mTOR is closely related to the tumor deterioration and poor survival time in meningioma [10]. Thus, investigating the expression and activation of OGN, mTOR, and NF2 signaling in meningioma cells will not only uncover key molecular mechanisms, but may also provide promising targets for the treatment of meningioma [8].

Chrysophanol is an anthraquinone with purported potent antitumor effects [11,12]. How chrysophanol exerts its anticancer effects in meningioma is unknown. To address this, we exposed cancer cells with increasing concentrations of chrysophanol in different periods. We supposed that chrysophanol could inhibit the growth of HBL-52 cells via decreasing cell proliferation and inducing apoptosis, and regulate the signaling transduction of OGN/mTOR and NF2 cascades. Therefore, this study assessed its effects on cell proliferation, cell viability, apoptosis, and signaling via OGN/mTOR and NF2 pathways.

## Materials and methods

### Cell culture

HBL-52 cells were obtained from Bafeier Biotechnology (Wuhan, China). Cells were cultured in DMEM medium (Sigma-Aldrich, MA, USA) supplemented with 10% fetal bovine serum and 1% streptomycin (100 μg/mL) and penicillin G (100 U/mL). Cells were maintained in an incubator at 37 °C with 5% CO_2_. The fourth to sixth generation of HBL-52 cells was used in the follow-up experiments, and each test repeats for three times.

### OGN overexpression and inhibition

HBL-52 cells were transfected with control siRNA (Lot#09212-212, Bafeier Biotechnology, Wuhan), OGN mimic (Sequence: 5’-GAAGGUGUAUGAGUA-3’, Lot#HSS210987, Bafeier Biotechnology, Wuhan), or OGN inhibitor (Sequence: 5’-GCGGCAUGUAUUGAG-3’, Lot#HSS123089, Bafeier Biotechnology, Wuhan) plasmids per the manufacturer’s instructions. Briefly, cells were cultured in a density of 4.0 × 10^5^ cells/well in 12-well plates for 12 h. Then 5 μL of plasmids and 500 μL of DMEM medium were mixed for 5 min; 5 μL of lipofectamine^TM^2000 and 500 μL of DMEM medium were also mixed for 5 min. The two mixtures were mixed for 20 min, then added and incubated cells. Cells transfecting with control siRNA, OGN mimic, and OGN inhibitor were grown for 24 h, rinsed with phosphate-buffered saline (PBS) and treated with chrysophanol.

### Cell viability assay

HBL-52 cells were grown in 96-well plates for overnight and cultured with varying doses of chrysophanol (0, 15, 30, 60, or 90 μM) for 12, 24, 48, and 72 h. At these time points, cell supernatant were removed, cultures were rinsed twice with PBS, and the CCK8 assay (Invitrogen Life Technologies, MA, USA) was performed based on the manufacturer’s instructions.

### Colony formation assay

HBL-52 cells were grown in 6-well plates for 24 h and incubated with increasing doses of chrysophanol (0, 15, 30, 60, and 90 μM) for 48 h. Cells were counted using a inverted microscope (OLYMPUS, IX73-A12FL/PH). Cell colonies were assessed by selecting 10 views and calculating the average number of cells.

### Histone DNA enzyme-linked immunosorbent assay (ELISA)

Cancer cells were grown in 6-well plates to a confluence of 60% and incubated for 48 h with different concentrations of chrysophanol (0, 15, 30, 60, and 90 μM). Then cells were lyzed, and recovered the supernatant after centrifugation. Bromodeoxyuridine (BrdU) and histone DNA levels were quantified using the respective ELISA kits per the manufacturers’ protocols. Optical density (OD) at 507 nm was determined by a microplate reader (Potenov, Beijing, China).

### Caspase-3 and −9 activity assays

Cells were grown in 6-well plates as above and incubated for 48 h with increasing doses of chrysophanol (0, 15, 30, 60, and 90 μM). Caspase activity was assayed using commercial kits (Abcam, UK).

### Annexin V/propidium iodide (PI) staining experiment

Treated cells were collected and adjusted to a density of 1 × 10^6^ cells/mL. Cell suspensions (0.5 mL) were stained with 1.25 μL Annexin V-FITC (Potenov, Beijing, China) at 37 °C in the dark for 10 min, and 10 μL of PI (Potenov, Beijing, China) was added for 10 min. Proportions of cells in different stages of cell cycle and apoptotic rates were estimated using flow cytometry (Beckman, USA).

### Western blotting

Protein from HBL-52 cells was extracted using RIPA buffer (Beyotime Biotechnology). Protein was quantified and equal amounts (50 μg) from each sample were resolved in 12% SDS-PAGE. Protein was transferred onto a nitrocellulose membrane (Invitrogen Life Technologies, MA, USA) before overnight incubation at 4 °C with primary antibodies (diluted 1:1000; Abcam, Cambridge, USA) against OGN, phosphorylated mTOR (p-mTOR, ab1098763), mTOR (ab2098461), NF2 (ab1128974), cleaved caspase-3 (ab1198329), cleaved caspase-9 (ab1232458), Bcl-2 (ab1123894), and Bax (ab1126478). Strips were then incubated for 2 h with horseradish peroxidase-conjugated goat anti-rabbit IgG (1:3000; Cell Signaling, Danvers, USA). Then strips were detected and analyzed to assess the relative expression levels of target proteins.

### Statistical analysis

All data were shown as average value± standard deviation. Student’s *t*-test was carried out to assess statistical differences in different groups, whereas ANOVA was performed to analyze the differences of multiple groups. Differences associated with *P*< 0.05 were regarded as significant.

## Results

### Chrysophanol suppresses growth of HBL-52 cells

Based on prior knowledge, chrysophanol may affect the proliferation and apoptosis of HBL-52 cells through the inhibition of OGN/mTOR pathway and the activation of NF2. Thus, we further explore the impacts of chrysophanol on the growth of malignant meningioma cultures, and observe its potential antitumor mechanism.

**Figure 1.**
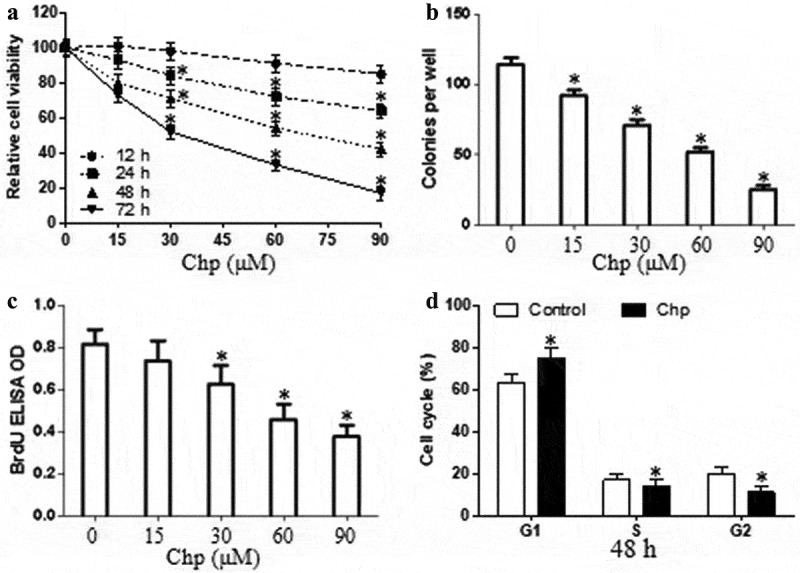
Chrysophanol (Chp) inhibits the growth of HBL-52 cells *in vitro*. Cells were treated with different concentrations of chrysophanol for the indicated periods, and (a) CCK8, (b) cell colony formation, and (c) bromodeoxyuridine (BrdU) assays were conducted. (d) The distribution of cells in different phases of the cell cycle was determined by flow cytometry after treatment with 60 μM chrysophanol for 48 h. *P< 0.05, compared with 0 μM chrysophanol group or control group

We determined whether chrysophanol affected HBL-52 cell proliferation using the CCK8 assay. Cultures were exposed to concentrations ranging from 2 μM (data not shown) to 90 μM for different periods. Chrysophanol inhibited proliferation of HBL-52 cells in a time- and concentration-dependence (*p*< 0.05, Figure 1a). These findings based on viability were confirmed in an assay based on colony formation (*p*< 0.05, Figure 1b). Treated cells also incorporated lower amounts of BrdU into the genome, indicating reduced proliferation (*p*< 0.05, Figure 1c). We then exposed cultures to 60 μM of chrysophanol for 48 h to assess the compound’s effects on the cell cycle (*p*< 0.05). We chose this concentration and time point because the relative cell viability is 53%. Chrysophanol incubation elevated the proportion of cells in G1 phase and reduced the proportion in S or G2 phases (Figure 1d).

### Chrysophanol induces apoptosis in HBL-52 cells

**Figure 2.**
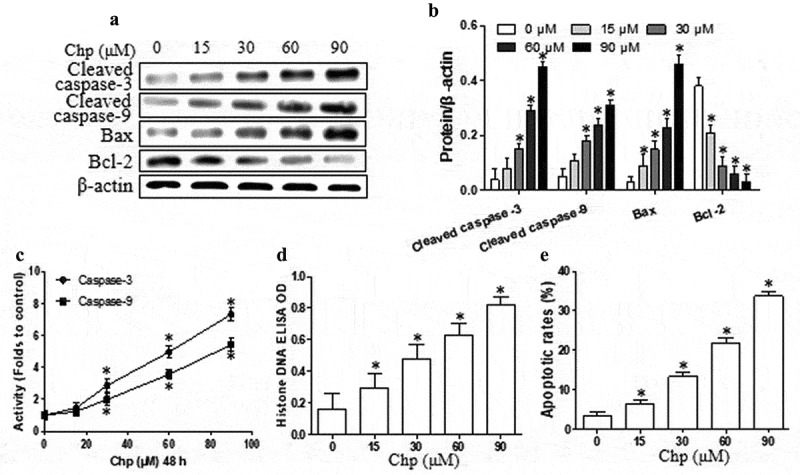
Chrysophanol (Chp) induces apoptosis of HBL-52 cells *in vitro*. Cells were treated with different concentrations of chrysophanol for 48 h. (a) Expression levels of caspase-3, caspase-9, Bax, and Bcl-2 were determined using western blot. (b) Densitometry of proteins from the experiments in panel A. (c) Activity of caspase-3 and −9. (d) Histone DNA levels. (e) Annexin V and propidium iodide staining, as captured by flow cytometry. *P< 0.05, compared with 0 μM chrysophanol

Next, we investigated whether and how chrysophanol induces apoptosis in HBL-52 cells. Treating HBL-52 cells with chrysophanol for 48 h significantly up-regulated cleaved caspase-3, cleaved caspase-9, and Bax protein, while dramatically down-regulating Bcl-2 (*p*< 0.05, Figure 2a and b). Interestingly, chrysophanol at concentrations as low as 15 µM appreciably affected Bax and Bcl-2 expression. Furthermore, chrysophanol increased the activity of caspase-3 and −9, as reflected in higher levels of histone DNA, in a dose-dependence (*p*< 0.05, Figure 2c and d). Treating cells with 90 μM chrysophanol for 48 h led to a 10-fold higher proportion of apoptotic cells than in untreated cultures (33.7% vs 3.3%, *p*< 0.05, Figure 2e).

### Chrysophanol reduces OGN/mTOR signaling but activates NF2 signaling in HBL-52 cells

**Figure 3.**
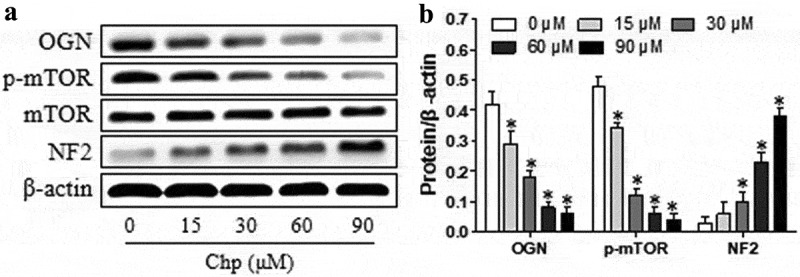
Chrysophanol (Chp) reduces OGN/mTOR signaling and activates NF2 signaling in HBL-52 cells *in vitro*. Cells were treated with different concentrations (0, 15, 30, 60, and 90 μM) of chrysophanol for 48 h. (a) Western blot of OGN, p-mTOR, total mTOR, and NF2. (b) Densitometry quantifying the relative levels in the experiments in panel A. *P< 0.05, compared with 0 μM chrysophanol

*OGN* is regarded as a novel oncogene in meningioma development.^8^ The OGN protein induces meningioma proliferation by down-regulating NF2 and activating mTOR signaling.^8^ Therefore, we used a western blot assay to evaluate the expressions of OGN, p-mTOR, and NF2 in cells after 48-h exposure to different concentrations of chrysophanol (*p*< 0.05, Figure 3). Treatment reduced levels of OGN and p-mTOR but increased NF2 levels in a concentration-dependence (*p*< 0.05). Our findings indicated that chrysophanol inhibits OGN/mTOR signaling but induces NF2 signaling in meningioma cells.

### OGN activates mTOR and down-regulates NF2

**Figure 4.**
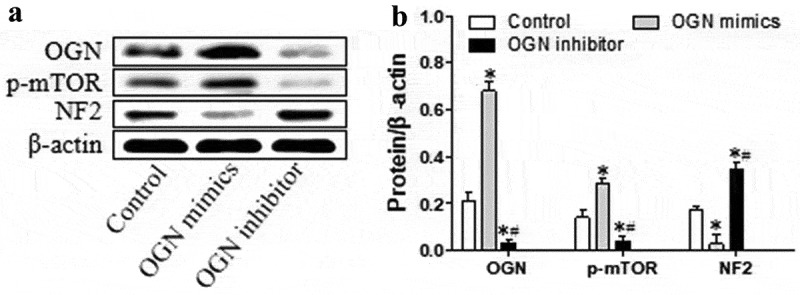
OGN induces activation of mTOR and down-regulates NF2 in HBL-52 cells *in vitro*. Cells were transfected for 48 h with plasmids (100 nM) overexpressing OGN mimics or inhibitors, then assayed. (a) Western blot of OGN, p-mTOR, and NF2. (b) Quantitation of the experiments in panel A. *P< 0.05, compared with 0 μM chrysophanol

OGN is known to interact with p-mTOR and NF2 during meningioma development, but how OGN affects these other proteins is not clear [8]. Therefore, we modified OGN expression and examined the effects on p-mTOR and NF2. HBL-52 meningioma cells overexpressing OGN showed increased levels of activated mTOR and down-regulated NF2 protein at 48 h (*p*< 0.05, Figure 4). Conversely, silence of OGN reduced levels of p-mTOR and up-regulated NF2 (*p*< 0.05, Figure 4). These findings suggest that OGN is an upstream factor to regulate mTOR and NF2 signaling.

### Chrysophanol regulates OGN, which in turn regulates signaling by mTOR and NF2

**Figure 5.**
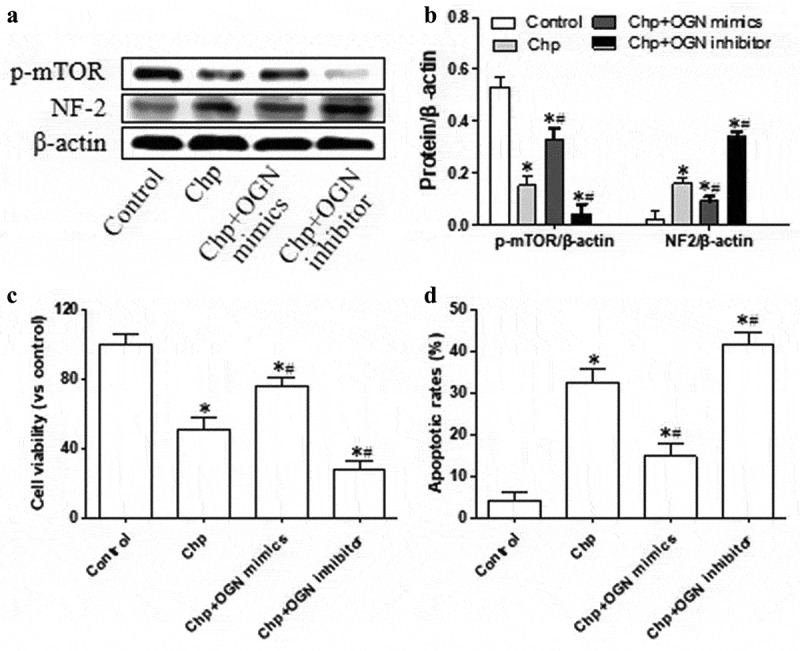
Chrysophanol acts via OGN to alter mTOR and NF2 signaling and growth of HBL-52 *in vitro*. Cells were transfected with plasmids encoding OGN mimetic or inhibitor, then treated with 60 µM chrysophanol for 48 h. (a) Western blot of p-mTOR and NF2. (b) Densitometry of the experiments in panel A. (c) Cell viability was analyzed by CCK8 assay. (d) Proportions of cells in apoptosis based on flow cytometry. *P< 0.05, compared with control group; ^#^*P*< 0.05, compared with chrysophanol group

Next we investigated whether the effects of chrysophanol on OGN may mediate its effects on levels of p-mTOR, expression of NF2, viability, and apoptosis in HBL-52 cells. Cells overexpressing OGN and treated with chrysophanol produced significantly higher levels of activated mTOR and significantly lower levels of NF2 than cells only treated with chrysophanol (*p*< 0.05, Figure 5). OGN overexpression also partially reversed the impacts of chrysophanol on cell viability and apoptosis (*p*< 0.05). Loss of OGN led to the opposite results. These experiments suggest that chrysophanol alters levels of p-mTOR and NF2 and cell growth by altering OGN expression.

## Discussion

Despite extensive observations about genomic and epigenomic maps have explored the pathogenesis and disease progression in meningioma, little is understood about the signaling pathways and key molecules involved [13–15]. OGN has been implicated in meningioma through effects on mTOR and NF2 signaling, but whether it may be a therapeutic target is unclear [16,17]. Here we provide evidence that chrysophanol suppresses the growth of HBL-52 cells by regulating the OGN cascade, implying its potential value as a drug target.

Chrysophanol belongs to a family of organic compounds with antitumor effects [18], and it suppresses the growth of multiple tumor cells through regulation of different signaling pathways. It has reported that chrysophanol has an anti-tumor role in the cell proliferation of lung cancer via mediating ROS/HIF-1a/VEGF signaling cascade [19]; additionally, it also suppresses cell growth, migration, and reactive oxygen species generation in oral cancer cell lines [20]. Moreover, it induces apoptosis of malignant optic nerve meningioma cells via inducing the caspase activation and targeting the MAPKs signaling cascade [21]. Similarly, our results in a menangioma cell type suggest that chrysophanol suppresses the proliferation of tumor cells while promoting their apoptosis. Our study goes further by suggesting that these antitumor effects begin with downregulation of OGN.

In colon cancer cells, chrysophanol blocks proliferation by inhibiting the mTOR pathway [22]. We also found that chrysophanol dose-dependently reduced proliferation of a meningioma cell line *in vitro*. This finding was based on the observation of decreases in cell viability, colony formation and BrdU+ incorporation. It is well known that Bcl-2/Bax ratio is a vital determinant of apoptosis induction [23–25]; our research demonstrated that chrysophanol induced Bax expression and inhibited Bcl-2 level, thus biasing the ratio toward cell death. This was further supported by increased expression and activity of pro-apoptotic caspase-3 and −9 in our experiments [26–30].

There is a obvious limitation in this study. We could not clearly demonstrate the interact of chrysophanol with OGN protein in malignant meningioma cells. Therefore, further studies should examine how chrysophanol affects the expression of OGN in malignant meningioma cells. Additionally, in our study, the potential of chrysophanol for treating malignant meningioma and other types of cancer can be verified and fully understood, improving its chances for clinical use.

## Conclusions

In summary, this present observation demonstrates the inhibitory effect of chrysophanol on malignant meningioma cells and its regulatory roles in the signaling transduction of OGN/mTOR and NF2 cascades. Our results should be verified and extended in other malignant meningioma cell lines and in preclinical models. OGN may be a useful target for treating this disease.

## Supplementary Material

Supplemental Material

## Highlights

• Chrysophanol suppresses the growth of meningioma cells.

• Chrysophanol inhibites OGN/mTOR signaling pathway.

• Chrysophanol activates NF2 signaling.
