# Supplementary figures and images for: Chrysophanol inhibits the osteoglycin/mTOR and activats NF2 signaling pathways to reduce viability and proliferation of malignant meningioma cells
Source: Bioengineered. 2021 Feb 23;12(1):755–62. doi: 10.1080/21655979.2021.1885864 (PMC8291820; doi:10.1080/21655979.2021.1885864)

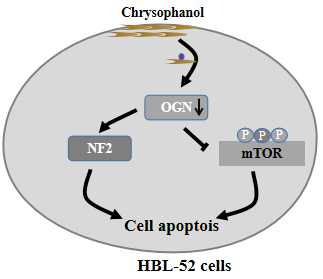

Supplement: Supplemental Material [file KBIE_A_1885864_SM0290.tif]
